# Simultaneous Sigmoid Volvulus and Small Bowel Obstruction: A Case Report

**DOI:** 10.7759/cureus.29338

**Published:** 2022-09-19

**Authors:** Isaac K Lee, Neng Zhong, Sana Aksas, Mohammad Masri

**Affiliations:** 1 College of Medicine, Dr. Kiran C. Patel College of Osteopathic Medicine, Nova Southeastern University, Fort Lauderdale, USA; 2 General Surgery, Larkin Community Hospital, Miami, USA; 3 General Surgery, University of Miami, Miami, USA; 4 Surgical Oncology, Larkin Community Hospital, Miami, USA

**Keywords:** small bowel, sigmoid, obstruction, volvulus, sbo, small bowel obstruction, sigmoid volvulus

## Abstract

Sigmoid volvulus and small bowel obstruction are typically thought to be separate clinical pathologies with distinct clinical features, diagnostic criteria, and treatment strategies. We present a rare case of simultaneous sigmoid volvulus and small bowel obstruction. To our knowledge, this is the first such case in literature and presented a unique set of challenges in regard to treatment and management. This case discusses a different approach to the surgical management of sigmoid volvulus and small bowel obstruction, which is markedly different from the expected and traditional surgical management of isolated sigmoid volvulus and small bowel obstruction.

## Introduction

Sigmoid volvulus

Sigmoid volvulus occurs when the sigmoid colon twists on itself, which can lead to large bowel obstruction and sigmoid colon ischemia [1]. Obstruction of the intestinal lumen and impairment of vascular perfusion occurs when the degree of torsion exceeds 180 and 360 degrees, respectively [2]. If left untreated, the volvulus can lead to perforation, which will result in colonic contents (i.e., stool, colonic bacteria, etc.) spilling into the abdomen.

Diagnostically, sigmoid volvulus can be identified by a classical “coffee-bean” sign on a plain abdominal X-ray [3]. Other imaging features that describe sigmoid volvulus include the ‘whirl sign,’ ‘split wall sign,’ and dilation of the sigmoid colon with a distal transition point [4]. From a clinical perspective, patients often present with the classic triad of abdominal pain, abdominal distension, and constipation [4]. The patient presented in this case displayed abdominal pain and distension without constipation.

Treatment of sigmoid volvulus is often achieved by flexible sigmoidoscopy detorsion, a Hartmann’s procedure, or resection with primary anastomosis, based on surgeon preference and clinical eligibility.

In patients who are hemodynamically stable and at high risk for surgical complications, sigmoid volvulus can be treated with flexible sigmoidoscopy; however, a study conducted by Ballantyne et al. demonstrated a 50% recurrence in sigmoid volvulus following flexible sigmoidoscopy detorsion [5]. For this patient, a 50% recurrence rate was too high for our liking. As such, we decided not to consider this option.

In patients with signs of peritonitis or perforation, detorsion of the volvulus can lead to reperfusion injury [2]. Consequently, instead of flexible sigmoidoscopy detorsion of the volvulus a Hartmann’s procedure or sigmoid colon resection with primary anastomosis should be performed. Hartmann’s procedure involves resection of the rectosigmoid colon and closure of the rectal stump with formation of an end colostomy [6]. On the other hand, the sigmoid colon can be resected and reconstructed with primary anastomosis, with or without proximal diversion. In the presence of hemodynamic instability, coagulopathy, acidosis, or hypothermia, Hartmann's procedure is preferred over primary anastomosis or flexible sigmoidoscopy [2].

Small bowel obstruction

Small bowel obstruction (SBO) is a common surgical emergency that can be caused by a multitude of etiologies. The most common cause of SBO in developed countries is intra-abdominal adhesions. Other common etiologies include incarcerated hernias, malignancy, inflammatory bowel disease, stool impaction, foreign bodies, and volvulus.

Diagnostically, the hallmark of a SBO is dilation of the small bowel, proximal to the site of obstruction, with decompression of the distal bowel with plain radiograph and/or CT imaging of the abdomen [7]. The extent of dilation should be out of proportion to the colon. Additionally, SBO can be described by the ‘stretch sign,’ which describes small amounts of gas separated by mucosal folds of the small bowel in a primarily fluid-filled bowel [7].

Initial management of SBO includes fluid resuscitation, pain control, antibiotics, and nasogastric (NG) decompression [8]. In some instances, NG decompression may serve as a conservative method for ileus and partial SBO. Indications for surgery also include whether the SBO is partial or complete and if it is strangulated or non-strangulated [8]. Surgical exploration in the setting of small bowel obstruction is indicated for suspected bowel compromise, such as bowel ischemia, necrosis, or perforation, or in treatment of a surgically correctable cause of SBO; however, adhesions, alone, are not an indication for surgery [9]. Radiologic signs such as free air on plain radiographs and closed-loop obstructions can also indicate bowel compromise.

## Case presentation

An 81-year-old black male with a past medical history of seizures, hypertension, asthma, unspecified psychiatric disorder, and dementia presented to the Emergency Department at Larkin Community Hospital in Miami, Florida via ambulance for abdominal pain, diarrhea, and vomiting.

He is a resident of an assisted living facility (ALF). He reports his symptoms started earlier the same day and rated the abdominal pain a six out of 10. He had approximately three episodes of non-bloody diarrhea but denied having any fever, cough, shortness of breath, chest pain, heart palpitations, or urinary symptoms. Physical examination was notable for abdominal distension, tenderness to deep palpation, and hypoactive bowel sounds with auscultation. There was no hepatomegaly or splenomegaly noted. Additionally, the CT scan of the abdomen revealed a possible bowel obstruction and a sigmoid volvulus.

**Figure 1:**
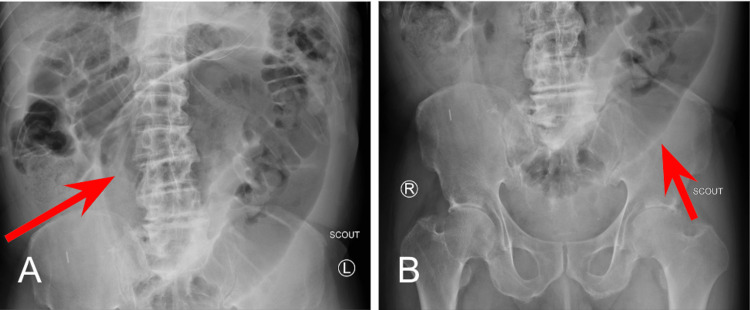
Plain Radiographs Prior Oral Contrast Administration 1A: Upper abdomen; 1B: Lower abdomen

A small bowel series was performed and revealed delayed transit of contrast through the small bowel and dilated loops of the large bowel without enteric contrast, which was concerning for obstruction. Plain radiographs of the upper and lower abdomen prior to the administration of oral contrast can be seen in Figure 1.

**Figure 2:**
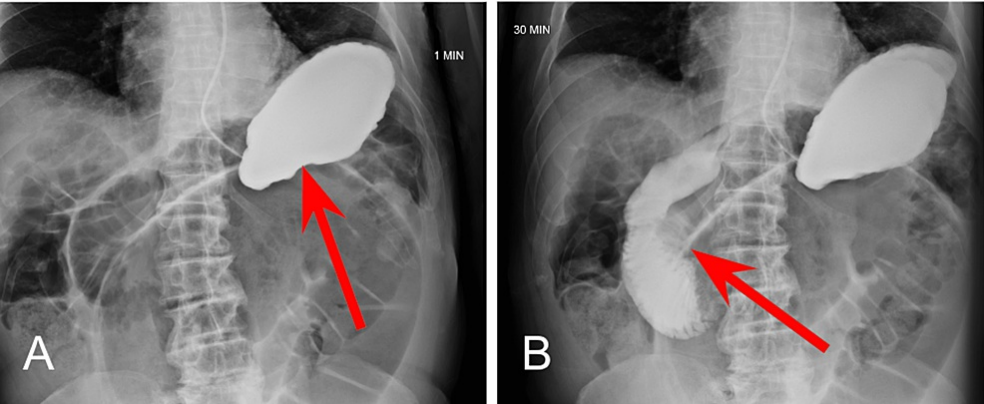
Small Bowel Series at One Minute and 30-Minutes 2A: One minute mark; 2B: 30-minute mark

At the 1 minute mark (Figure 2A), opacification of the stomach following administration of contrast material via NG tube can be noted. At the 30-minute mark (Figure 2B), normal transit of contrast into the proximal small bowel can be visualized within the duodenum.

**Figure 3:**
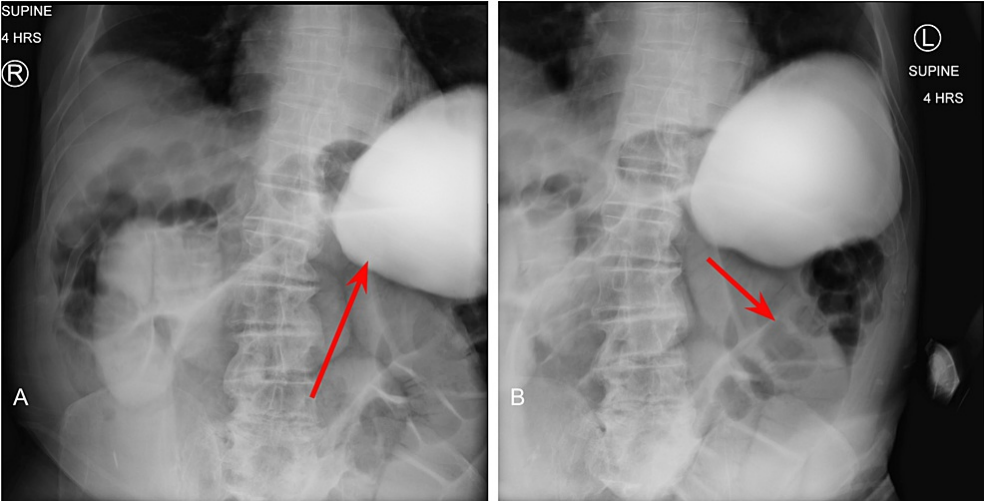
Small Bowel Series at Four Hours 3A: Oral contrast in the mid-to-distal small bowel; 3B: dilated loops of bowel

At the four-hour mark, enteric contrast is noted within the mid to distal small bowel (Figure 3A), with dilated loops of large bowel without contrast distally (Figure 3B). Without passage of oral contrast at the four hour mark, our suspicion for SBO located at the distal jejunum and proximal ileum was confirmed.

The patient was subsequently diagnosed in the emergency department with small bowel obstruction (SBO) and lactic acidemia (Table 1). He was admitted for further medical treatment and surgical evaluation. Upon admission, he was placed on a nothing-by-mouth (NPO) diet, and an NG tube and foley catheter were inserted. Immediately after placement of the NG tube, approximately 1,000mL of gastric material was retrieved. The patient was resuscitated overnight with IV fluids, and his lactic acid decreased from 2.2mg/dL to 0.8mg/dL. 

**Table 1:** Laboratory Data POD: Postoperative Day; (H): Higher than normal values; (L): Lower than normal values; uL: microliter; mcL: microliter; g/dL: grams per deciliter; mmol/L: millimole per liter; U/L: units per liter; mg/dL: milligrams per deciliter; mEq/L: milliequivalent per liter; BUN/CREA: blood urea nitrogen to creatinine ratio; mOsm/L: milliosmoles per liter

Variable	Reference Range	On Initial Presentation	POD #1	POD #3	POD #6
White-Cell Count (10^3^/uL)	5.87-11.5	12.97 (H)	8.30	10.18	7.12
Differential Count					
Neutrophils (10^3^/mcL)	1.78-5.38	11.29 (H)	7.20	8.37	4.42
Lymphocytes (10^3^/mcL)	1.32-3.57	0.79 (L)	0.43 (L)	0.65 (L)	0.99 (L)
Monocytes (10^3^/mcL)	0.30-0.82	0.80	0.64	0.69	0.91 (H)
Eosinophils (10^3^/mcL)	0.04-0.54	0.00 (L)	0.00 (L)	0.32	0.29
Platelet Count (10^3^/uL)	150-450	209.0	149.0 (L)	135.0 (L)	187.0
Red-Cell Count (10^6^/mcL)	4.47-5.80	4.19 (L)	3.68 (L)	3.14 (L)	3.21 (L)
Hemoglobin (g/dL)	12.1-16.1	12.8	11.4 (L)	9.5 (L)	9.7 (L)
Hematocrit (%)	36.0-47.7	36.8	32.4 (L)	28.4 (L)	28.2 (L)
Lactic Acid (mmol/L)	0.55-1.99	2.2 (H)	-	0.80	-
Alanine aminotransferase (U/L)	7-40	40	33	23	73 (H)
Aspartate aminotransferase (U/L)	7-30	44 (H)	73 (H)	32	76 (H)
Alkaline phosphatase (U/L)	31-110	101	58	50	82
Amylase (U/L)	31-115	126 (H)	-	-	-
Lipase (U/L)	11-104	55	-	-	-
Bilirubin, Total (mg/dL)	0.11-0.93	1.43 (H)	1.50 (H)	0.63	0.68
Sodium (mEq/L)	135-145	138	138	137	138
Potassium (mEq/L)	3.4-4.9	4.3	3.8	3.5	3.7
Chloride (mEq/L)	95-107	101	103	102	102
Calcium (mg/dL)	8.3-10.1	10.2 (H)	8.0 (L)	7.6 (L)	8.2 (L)
Carbon dioxide (mEq/L)	22-31	25	27	30	31
Blood urea nitrogen (mg/dL)	6-20	27 (H)	23 (H)	-	17
Creatinine, serum (mg/dL)	0.45-1.29	1.43 (H)	1.34 (H)	-	-
BUN/CREA	7.0-25.0	19	17	11	18
Osmolality, serum (mOsm/L)	261-280	281.9 (H)	281.8 (H)	-	278.1
Total protein (g/dL)	5.9-7.9	7.8	5.1 (L)	4.4 (L)	4.9 (L)
Albumin (g/dL)	3.5-5.1	4.7	3.1 (L)	2.6 (L)	2.9 (L)
Glucose (mg/dL)	75-105	119 (H)	143 (H)	120 (H)	114 (H)

The following morning, he continued to have abdominal distention and a high NG tube output. An emergent exploratory laparotomy was scheduled for lysis of adhesions and possible bowel resection.

Physical exam

*General:* The patient was awake and alert, with no apparent distress.

*Cardiovascular:* S1 and S2 heart sound present, regular rate and rhythm, with no palpable heaves, thrills, or lifts. Pulses were normal, and the blood pressure did not differ substantially between the two arms or between the two legs. No notable edema. No jugular vein distension.

*Respiratory*: Lungs were clear to auscultation, bilaterally, with bilateral symmetric chest expansion. No Ronchi, rales, or wheezing. No use of accessory muscles, no grunting, no evidence of nasal flaring, no paradoxical movements, no prolonged exhalations or shallow respirations, no pursed lip breathing, no costal retractions, and no tachypnea.

*Abdominal/GI:* Surgical scar present approximately 10cm, midline. Physical examination was notable for abdominal distension, tenderness to deep palpation, and hypoactive bowel sounds with auscultation. There was no hepatomegaly or splenomegaly noted. 

## Discussion

Differential diagnosis

The diagnosis of small bowel obstruction, secondary to abdominal adhesions, was high on the list of differential diagnoses based on the clinical presentation and previous abdominal scar; however, several aspects of the patient’s past medical history and clinical presentation still provided a few other diagnoses to consider. Factors that were considered for other diagnoses are listed below (Table 2). Nonetheless, our initial diagnosis of small bowel obstruction was confirmed by the small bowel series. Additionally, the CT scan conducted on the day of admission discovered a possible sigmoid volvulus.

**Table 2:** Features of This Case Relevant to the Possible Diagnoses Risk factors, clinical features, and laboratory findings of this patient's case corresponding with other differential diagnoses.

Diagnosis	Risk Factors	Clinical Features	Laboratory Features
Small Bowel Obstruction	Previous abdominal surgery indicated by abdominal scar	Nausea and/or vomiting; abdominal pain; abdominal distension; abdominal tenderness to palpation; hypoactive bowel sounds	Leukocytosis; neutrophilia; lactic acidosis
				Sigmoid Volvulus	African American descent; age >70 years old; male gender; neuropsychiatric disorders; previous abdominal procedure	Abdominal pain, continuous and severe; nausea and/or vomiting; abdominal distension; constipation; tympanitis	
				Acute Pancreatitis	Male gender	Acute onset of epigastric pain; nausea and/or vomiting; abdominal tenderness to palpation; abdominal distension; hypoactive bowel sounds	Elevated serum amylase
				Mesenteric Ischemia		Abdominal pain; abdominal distension	Leukocytosis; lactic acidosis; elevated serum amylase

Preoperative diagnosis

Simultaneous small bowel obstruction, secondary to abdominal adhesions with sigmoid volvulus

Discussion of preoperative management

In regards to the patient’s SBO, his persistent signs and symptoms, despite adequate intravenous fluids, antibiotics, and adequate pain control, warranted continued consideration for surgical management. Ultimately, the high NG tube output of approximately 1,000mL of bowel contents was one of the final factors that indicated surgical management of his SBO. The plan was to perform a small bowel resection with primary anastomosis.

In regards to his sigmoid volvulus, the signs of peritonitis associated with sigmoid volvulus and lactic acidosis indicated the likely surgical procedure for this patient to be Hartmann’s procedure.

After consent was obtained for exploratory laparotomy with lysis of adhesions and possible bowel resection and colostomy, he was kept NPO, and a type and screen were performed. Preoperative antibiotics were administered in the form of 1 gram of intravenous Rocephin. Due to his presentation and urgency for surgical management, preoperative oral laxatives for bowel preparation were not achieved.

Discussion of surgical management

Upon exploration, the patient was confirmed to have evidence of both small and large bowel obstruction, as well as sigmoid volvulus. Extensive adhesions, a small bowel stricture, and two abdominal foreign bodies were also found. During the operation, dissection of the adhesions occupied approximately 66% of the total operative time.

Sigmoid Volvulus

Following adhesion dissection, the sigmoid colon was found to be volvulus around the inferior mesenteric artery, torsed twice. After the detorsion of the sigmoid volvulus, it was tested for viability. Although there was significant enlargement and dilation of the proximal and distal segments of the sigmoid colon to the volvulus, there was no evidence of colonic ischemia, perforation, or gangrene necrosis. There was also no sign of stricture or tumor distally at the rectum or proximal to the volvulus. The sigmoid volvulus was resolved, and a sigmoidectomy with primary side-to-side and functional end-to-end anastomosis was performed. The mesentery was layered on top of the anastomosis to improve healing.

Following the resolution of the sigmoid volvulus and subsequent sigmoidectomy, we ran the bowel for other pathologies. While running the bowel, we encountered multiple areas of adhesions, which were carefully lysed with a combination of blunt dissection, finger dissection, and sharp dissection using Bovie, Metzenbaum scissors, and the Harmonic scalpel. Upon reaching the ileum, we found evidence of a previous anastomosis, approximately 10 cm in length, which was created in an antiperistaltic fashion. We also discovered a stricture and two blind ending loops at this location.

Small Bowel Obstruction

While observing the peristalsis of the succus when running the bowel, we found an area of stricture at the distal jejunum and ileum, along with difficulty for the succus to pass through distally into the cecum and ascending colon. This stricture, which was caused primarily by the antiperistaltic segment, was noted to be the cause of the SBO, and we also identified two small perforations in the jejunum. We decided it was in his best interest to resect this portion of the small bowel. We started the bowel resection at the previous ileal anastomosis. Small bowel resection was performed with primary side-to-side, and functional end-to-end anastomosis was performed. The mesentery was then approximated and sutured to avoid internal herniation.

Abdominal Adhesions

We then turned our attention to the adhesions of the abdominal wall. Upon debridement of the abdominal wall, we discovered two foreign bodies that were hard and topically ossified. The foreign bodies were resected and sent to pathology.

Closing

We confirmed, through manual palpation, placement of the NG tube in the stomach. We placed a Jackson-Pratt (JP) drain in the pelvis to protect the sigmoid anastomosis. After adequate irrigation of the abdomen and confirmation that there was no bleeding, the abdominal fascia was closed. The subcutaneous tissue was irrigated, and the skin was closed with staples. The abdomen was cleaned and sterile dressings and an abdominal binder were applied.

Postoperative diagnoses

Following completion of the surgery, the patient's final diagnoses included: an acute abdomen, complete small and large bowel obstruction, sigmoid volvulus, extensive adhesions, small-bowel stricture, and removal of two abdominal foreign bodies.

Challenges to this case

Bowel Preparation

Typically, the common practices for bowel preparation include oral laxatives, preoperative oral antibiotics, and intravenous antibiotic prophylaxis. Due to the patient’s acute presentation, we were unable to perform adequate bowel preparation for surgery. Although preoperative antibiotics were given, we were unable to administer any laxatives to evacuate the bowel in preparation for surgery.

In a retrospective study conducted by Shahmoradi et. al, researchers concluded there was no significant difference in mortality, anastomosis leakage, prolonged ileus, bleeding, surgical site infection, or fascial dehiscence in an unprepped bowel between a Hartmann’s procedure and primary anastomosis [10]. Consequently, we felt confident and comfortable in our approach to performing a primary anastomosis despite having an unprepped bowel.

Small Bowel Obstruction (SBO)

Small bowel obstructions are commonly caused by scar tissue or adhesions, hernias, or cancer. During the procedure, we discovered evidence of previous abdominal surgery and anastomosis while running the bowel. Specifically, we identified an antiperistaltic segment of the bowel at the distal jejunum and ileum where the previous anastomosis had been performed. We believed this to be the most likely cause of SBO. However, we were unable to rule out other possible causes of SBO, such as adhesions or even the sigmoid volvulus.

Since this is the first case in the literature of a patient with simultaneous small bowel obstruction and sigmoid volvulus, although we highly suspect the antiperistaltic segment to be the primary cause of SBO, it is still worth noting that the SBO could have been caused by either the antiperistaltic segment and the sigmoid volvulus, or both simultaneously.

Before surgery, we attempted to conservatively manage his SBO, which involved NG tube placement. NG tube placement allows for the small bowel to rest, return to normal size, and hopefully resolve any obstructions that may be caused by adhesions from the previous surgery [11]. However, after failed management with NG tube decompression, we decided to operatively resolve the obstruction. 

Traditionally, surgical management of small bowel obstruction, secondary to adhesions, involves lysis of abdominal adhesions [11]. While this was our initial surgical plan, a unique obstacle we encountered during the procedure was the two perforations that were identified in the jejunum at the site of the previous anastomosis. When discussing surgical management of an anastomotic leak, as in this case, we considered several different options. In the setting of gross peritonitis, it is recommended that a washout of the abdomen be performed, followed by proximal diversion of the anastomosis and drainage of the anastomotic area. Fortunately, there was no gross peritonitis.

However, a study conducted by Murrell and Stamos discovered that a takedown of a leaking anastomosis can likely result in a permanent ostomy for many patients [12]. With this in mind, although there was no evidence of gross peritonitis, we had to consider performing a resection of the antiperistaltic segment, including the region of anastomosis leak, followed by an end ileostomy.

When discussing what would be best for this patient and considering his postoperative implications, we decided to forego resection followed by end ileostomy and performed a small bowel resection followed by primary anastomosis instead. Given his past medical history of seizures, unspecified psychiatric disorders, dementia, and residence in an ALF, we concluded that the management of an ileostomy bag would be too cumbersome for both the patient and the ALF staff. It would also diminish his quality of life. Additionally, given his history, an ileostomy bag places him at higher risk of complications associated with the care of the stoma and ileostomy bag maintenance, such as infection, stoma stricture, bleeding, and general hygiene. For these reasons, we decided bowel resection followed by primary anastomosis would be most beneficial, while also maximizing his quality of life.

In deciding to perform another resection and primary anastomosis, we posed the risk of an anastomotic leak, mesenteric entrapment, and anastomotic stricture, to name a few [13]. In all instances, there was the risk of anastomotic failure, which would have required another surgical operation that would result in an ileostomy anyway. However, when comparing his potential quality of life and postoperative indications for ileostomy vs. primary anastomosis. We elected to perform the primary anastomosis despite the recommendations found in the literature for traditional surgical management of reoperation for anastomotic failure. Fortunately, he tolerated the primary anastomosis well and did not exhibit any signs of anastomotic failure at his follow-up outpatient appointment approximately 25 days later.

Sigmoid Volvulus

When discussing the surgical management for his sigmoid volvulus, Hartmann's Procedure is indicated in the presence of hemodynamic instability, coagulopathy, acidosis, or hypothermia. Given his presentation of persistent abdominal pain and evidence of significant bowel obstruction and lactic acidemia, traditional surgical management of the sigmoid volvulus via Hartmann’s procedure was warranted. However, for the same implications and consequences mentioned previously when discussing surgical management of his SBO through end ileostomy, we discussed the benefits and consequences of performing a primary anastomosis versus Hartmann’s procedure for his sigmoid volvulus. When evaluating functionality and quality of life, we wanted to explore the option of performing a primary anastomosis in the management of his sigmoid volvulus.

A prospective randomized study by Sasaki et al. discovered improved healing in patients who underwent a primary anastomosis between two healthy segments of the bowel compared to resection followed by end-colostomy formation [14]. In evaluating the benefits and consequences of colostomy versus anastomosis, James et al. concluded the leak rate and mortality rate of a segmental resection with colocolonic anastomosis to be 2.7% and 1%, respectively [15]. The minimal incidence of mortality and anastomosis leak was compelling enough to determine primary anastomosis to be a safe procedure. 

Given the studies mentioned and the overall outcome of our patient, we feel confident and comfortable in our decision to repair both the SBO and sigmoid volvulus, with resection and primary anastomosis, as opposed to the traditional resection and end ileostomy and colostomy, respectively. During his outpatient follow-up appointment, he returned to baseline mentation and function and was without pain, discomfort, or any other clinical manifestations of his initial diagnosis.

Prolonged Ileus

This patient’s postoperative course was complicated by a prolonged postoperative ileus (POI) that lasted approximately 17 days. When determining the cause of the POI, we considered a multitude of possible etiologies for which the criteria by which we were able to rule out some causes are listed in Table 3 [16]. While it is unclear when the gastrointestinal tract ought to resume motility, research has concluded that the colon, which is typically the last portion of the gastrointestinal tract to resume motility, typically resumes normal activity at approximately 72 hours. Consequently, any POI lasting longer than 72 hours can be considered a pathological ileus. One of the primary complications commonly associated with prolonged POI, which we wanted to avoid, was a prolonged hospital stay. Especially with the Coronavirus-19 (COVID-19) pandemic, we wanted to quickly discharge him to limit his exposure and risk for contracting of COVID-19 infection. Additionally, prolonged POI poses risks and complications for increased healthcare costs, electrolyte derangements, malnutrition, and poor patient satisfaction [16]. These are all complications we hoped to avoid as much as possible.

**Table 3:** Suspected Etiologies of Postoperative Ileus Possible etiologies for the patient's postoperative ileus relative to this case. Rational for whether or not certain etiologies could be ruled out is also listed.

Etiologies	Evaluation Criteria and Conclusion
Interruption of the gastrointestinal continuity or manipulation of the bowel	Most likely etiology per surgical history, was not ruled out
Anesthesia and/or analgesic medication	Possible etiology, was not ruled out
Psychiatric medications	Possible etiology, was not ruled out
Immobility	[RULED OUT] per adequate ambulation with physical therapy
Electrolyte imbalance, especially hypokalemia	[RULED OUT] per lab results (See Table 1)
Intraabdominal hematoma	[RULED OUT] per CT Scan of the abdomen Post-op Day #8
Intraabdominal infection or sepsis	[RULED OUT] per lab results (See Table 1)
Severe pain	[RULED OUT] per patient, he was not experiencing severe pain and adequate analgesic medications were administered

Traditional treatment of POI often involves a multimodal approach. For example, Basse et al. discovered a multimodal approach involving epidural analgesia, early oral nutrition and mobilization, cisapride, a gastrokinetic agent, and laxative treatment with magnesia to be effective within 48 hours of colonic resection [17]. Other modalities for POI treatment include NG tube intubation, gum chewing, and laxative administration [18]. During the first week of his postoperative recovery, we decided to manage his POI conservatively with a variation of the multimodal approach suggested by Basse et al. by administering metoclopramide, along with early oral feeding and mobilization.

**Figure 4:**
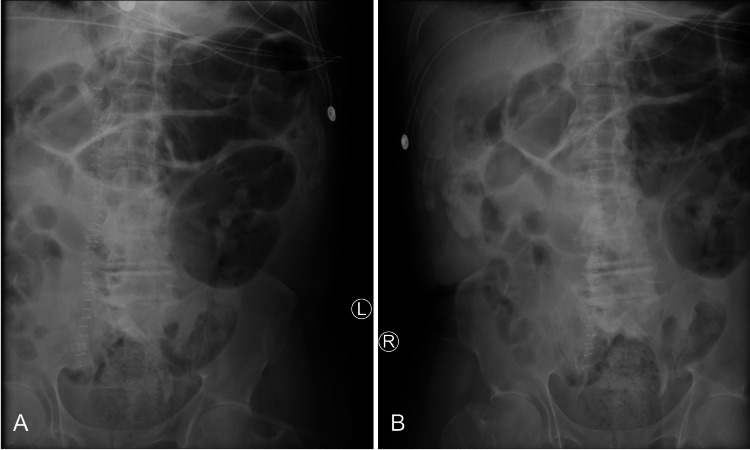
Kidney, Ureter, and Bladde (KUB) Plain Radiograph Kidney, Ureter, and Bladder X-Ray of the left and right abdomen (A and B). Multiple loops of prominent gas-filled small bowel without evidence of high-grade obstruction. Residual changes and material at the right abdomen and left lower quadrant. Nonspecific but nonobstructive bowel gas pattern with postoperative changes.

His POI was thought to have resolved after having one bowel movement approximately one week into his postoperative recovery; however, the following day, he presented with nausea, vomiting, tachycardia, and abdominal distension. A kidney, ureter, and bladder (KUB) plain radiograph was ordered, and re-demonstrated signs of ileus without perforation (Figure 4). Given the patient’s previous history of abdominal surgeries, we suspected another bowel obstruction, secondary to abdominal adhesions, as the cause of his prolonged ileus. We also considered a failed anastomosis. We discussed the possibility of performing another operation to identify and lyse any remaining adhesions, along with a possible correction of the resections and anastomoses we performed with end ileostomy and colostomy as traditionally indicated.

In determining whether another operation was warranted to search for another cause of prolonged ileus, we placed an NG tube with suction. We immediately retrieved approximately 1,000 mL of gastric fluid. Under suspicion of another possible obstruction or failed anastomosis from the initial surgery, we order a CT scan of the abdomen with contrast. Fortunately, the CT scan of the abdomen demonstrated contrast passing through the entire small and large bowel until the descending colon, thus excluding a complete bowel obstruction and/or failed anastomosis as the cause of prolonged ileus.

At this time, we diagnosed him with prolonged postoperative ileus and multifactorial gastroparesis, secondary to multiple antipsychotic medications. We requested a psychiatry consultation to discuss psychiatric medications possibly interfering with bowel movements. Upon further investigation, we suspected multifactorial gastroparesis, likely caused by the long history of antipsychotic medication use, as the primary culprit for the POI.

Following adequate medication reconciliation by psychiatry, we elected to try another trial of metoclopramide to stimulate gastrointestinal activity, along with the addition of oral Erythromycin in an attempt to resolve the POI. A common side effect of erythromycin is an increase in the number and force of gastric contractions [19]. For this reason, we administered erythromycin to address his diagnosis of multifactorial gastroparesis and to encourage gastric contractions and motility. We decided to allow time for conservative management and wait to see if the combination of metoclopramide and erythromycin would resolve the multifactorial gastroparesis and prolonged ileus.

Given the patient’s history of failed anastomosis and previous abdominal surgery, we strongly considered reoperation once the prolonged ileus was considered pathological, not physiological. However, we also wanted to provide him a little more time for his ileus to resolve than typically indicated. Given his past surgical history we considered the fact that his POI may take slightly longer to resolve in comparison to a virgin abdomen without a surgical history. Additionally, given the initial bowel movement he had approximately one week after surgery, we were not eager to take him back into surgery. We decided to remain as conservative as possible to try and give the anastomosis the opportunity to function.

We decided to continue a multimodal approach as recommended by Basse et al. Although we did not follow a specific regimen, we incorporated a multimodal approach which included metoclopramide, erythromycin, and early oral feeding and mobilization, along with adequate medication reconciliation given our suspicion for multifactorial gastroparesis. We ultimately hoped to delay and avoid operating on him again to resolve the POI.

After approximately 20 days following surgery, the patient had four total bowel movements within 24 hours. We advanced his diet from clear liquids to a soft diet, which he tolerated well. We considered the POI was resolved and continued to monitor his progression. While we are still unclear about the exact etiology of his POI, we suspect the prolonged POI was more a result of multifactorial gastroparesis, secondary to chronic antipsychotic medication use, instead of the surgical procedure itself. Fortunately, our medical and surgical decision not to reoperate was appropriate as the POI eventually resolved with conservative management, without any potentially fatal events.

He was discharged the following day and instructed to follow-up in two weeks. He was seen by Dr. Masri for his two-week follow-up appointment at the outpatient clinic. He was doing fine, having normal bowel movements, and tolerating his diet well. At this time, we signed off on the patient, determined the SBO, sigmoid volvulus, and postoperative ileus to be resolved, and will see him as needed moving forward.

## Conclusions

To our knowledge, this is the first case in literature of a simultaneous small bowel obstruction and sigmoid volvulus. One particular question we considered was if the sigmoid volvulus could have been the cause of the small bowel obstruction. Unfortunately, we were unable to answer this question. Additionally, all clinical indications pointed towards a small bowel resection with end ileostomy and colonic resection with end colostomy for the small bowel obstruction and sigmoid volvulus, respectively. While this was part of our surgical plan, when considering the patient’s medical and psychiatric history, living situation, and quality of life, we decided to pursue a more cavalier approach and attempt a small bowel resection with primary anastomosis and colon resection with primary anastomosis instead. 

One consequence or complication we were prepared to face was the need to operate on him again due to failed anastomoses, which would have ended with the end of the ileostomy and colostomy as surgically indicated. This was a risk we were prepared to take. We decided that if our plan to perform resection with primary anastomosis was successful, we would be happy with our surgical decision. On the other hand, if the surgical attempt for resection and primary anastomosis failed and warranted another procedure only to end with an end ileostomy and colostomy, we were still content with the attempt we would have taken to try to maximize his quality of life. Fortunately, our surgical decision was successful in the end, as he tolerated the resection and primary anastomosis well and, as far as we know, has not experienced any adverse side effects or complications from our surgical management. Despite the patient’s previous surgeries or not, the decision to perform the surgical procedure that will maximize, his quality of life with limited or reduced risks should always be considered. Regardless of the clinical or surgical indications, an experienced surgeon ought to exercise their professional judgment to take an alternative approach as long as the patient's best interest is in mind and the approach is safe for the patient as well. Additionally, despite the surgical aspects of this case, other medical and psychiatric diagnoses should always be considered when possible and appropriate. It cannot be definitively concluded whether or not his history of chronic antipsychotic medications was the etiology for prolonged postoperative ileus and multifactorial gastroparesis; however, given the resolution of the POI shortly after psychiatric medication reconciliation was performed, we highly suspect this to have played a role in his prolonged POI.

## References

[1] Baiu I, Shelton A (2019). Sigmoid volvulus. JAMA.

[2] Hodin RA (2022). UpToDate: Sigmoid volvulus. UpToDate, 24 June.

[3] Singh G, Gupta SK, Gupta S (1985). Simultaneous occurrence of sigmoid and cecal volvulus. Dis Colon Rectum.

[4] Bhattacharya S, Skole K (2017). Sigmoid volvulus: Diagnosis and management of a rare. Am J Gastroenterol.

[5] Ballantyne GH, Brandner MD, Beart RW Jr, Ilstrup DM (1985). Volvulus of the colon. Incidence and mortality. Ann Surg.

[6] Hallam S, Mothe BS, Tirumulaju R (2018). Hartmann's procedure, reversal and rate of stoma-free survival. Ann R Coll Surg Engl.

[7] Paulson EK, Thompson WM (2015). Review of small-bowel obstruction: the diagnosis and when to worry. Radiology.

[8] Schick MA, Kashyap S, Meseeha M (2022). Small Bowel Obstruction. https://pubmed.ncbi.nlm.nih.gov/28846346/.

[9] Bordeianou L, Daniel DY (2022). UpToDate: Management of small bowel obstruction in adults. UpToDate, 5 Feb.

[10] Kazem Shahmoradi M, Khoshdani Farahani P, Sharifian M (2021). Evaluating outcomes of primary anastomosis versus Hartmann's procedure in sigmoid volvulus: A retrospective-cohort study. Ann Med Surg (Lond).

[11] Baiu I, Hawn MT (2018). Small bowel obstruction. JAMA.

[12] Murrell ZA, Stamos MJ (2006). Reoperation for anastomotic failure. Clin Colon Rectal Surg.

[13] Davis B, Rivadeneira DE (2013). Complications of colorectal anastomoses: leaks, strictures, and bleeding. Surg Clin North Am.

[14] Sasaki LS, Allaben RD, Golwala R, Mittal VK (1995). Primary repair of colon injuries: a prospective randomized study. J Trauma.

[15] Murray JA, Demetriades D, Colson M (1999). Colonic resection in trauma: colostomy versus anastomosis. J Trauma.

[16] Buchanan L, Tuma F (2022). Postoperative Ileus. https://www.ncbi.nlm.nih.gov/books/NBK560780/.

[17] Basse L, Madsen JL, Kehlet H (2001). Normal gastrointestinal transit after colonic resection using epidural analgesia, enforced oral nutrition and laxative. Br J Surg.

[18] Luckey A, Livingston E, Taché Y (2003). Mechanisms and treatment of postoperative ileus. Arch Surg.

[19] King JE (2007). Why use erythromycin to manage gastroparesis?. Nursing.

